# Effects of baicalein with memantine on aluminium chloride-induced neurotoxicity in Wistar rats

**DOI:** 10.3389/fphar.2023.1034620

**Published:** 2023-02-22

**Authors:** Ratnakar Jadhav, Yogesh A. Kulkarni

**Affiliations:** Shobhaben Pratapbhai Patel School of Pharmacy & Technology Management, SVKM’s NMIMS, Mumbai, India

**Keywords:** baicalein, AlCl3, β-Amyloid, BDNF, oxidative stress, neurodegeneration, Alzheimer’s disease

## Abstract

Alzheimer’s disease is a progressive neurodegenerative condition. It is one of the most common 28 forms of dementia accounting for 60–80% of people suffering from dementia. There are very few medications that are approved for the treatment of Alzheimer’s disease. Baicalein, belonging to the flavone subclass of flavonoids, has been reported to have a neuroprotective effect by reducing oxidative stress and neuroinflammation, inhibiting the AChE enzyme, and reducing amyloid protein aggregation and toxicity. Memantine is one of the most important drugs used for treating Alzheimer’s disease. The purpose of this work was to study the effect of baicalein with memantine on aluminum chloride-induced neurotoxicity in Wistar rats. Aluminum chloride (100 mg/kg p.o.) was administered for 42 days in male Wistar rats to induce neurotoxicity. Baicalein alone (10 mg/kg) and a combination of baicalein (5 mg/kg and 10 mg/kg) with memantine (20 mg/kg) were administered for 42 days. Treatment of baicalein with memantine showed significant improvement in behavioral parameters. The combination reduced oxidative stress and the formation of β-Amyloid plaques and increased brain-derived neurotrophic factor (BDNF) expression. Based on findings, it can be concluded that treatment with baicalein and memantine may slow the progression of neurodegeneration in rats.

## 1 Introduction

Alzheimer’s disease (AD), the most prevalent form of dementia, is a progressive, multifaceted neurodegenerative disorder. It is estimated that around 55 million people are living with Alzheimer’s and other dementias, and that number will rise to 152.8 million by 2050 (AAIC, 2021).

AD is characterized by the accumulation of intracellular neurofibrillary tangles (NFT) and extracellular amyloidal beta (Aβ) plaques (Kumar et al., 2015). Aβ plaques activate microglia, leading to increased oxidative stress and local inflammatory response, both of which contribute to neurotoxicity. Neuronal cell death leads to a deficit in acetylcholine (Ach), a neurotransmitter involved in memory. Active oxygen causes direct neuronal damage by increasing intracellular Ca^2+^ and inflammation. The production of cytokines as a result of inflammatory responses inhibits brain-derived neurotrophic factor (BDNF) (Hachisu et al., 2015; Tiwari et al., 2019).

The current treatment of Alzheimer’s disease includes the use of cholinesterase inhibitors (CIs) for mild to moderate symptoms and memantine, an N-methyl-D-aspartate (NMDA) receptor antagonist, for moderate to severe AD symptoms. The FDA has approved a combination of CIs and NMDA receptor antagonists for the treatment of moderate to severe AD symptoms. However, the literature indicates side effects of the therapy, such as nausea, vomiting, diarrhea, abdominal cramps, etc (Yiannopoulou and Papageorgiou, 2013). As the current treatment options are limited and have side effects, there is an unmet need to explore new treatment options for AD.

With Alzheimer’s disease being a multifactorial disorder, the requirement for developing multi-target drugs with fewer side effects has become an urgent “medical need.” Natural compounds have received an increase in interest in recent years due to their efficacy and safety profile. Flavonoids, one of the largest groups of natural products, have attracted attention because of their extensive therapeutic effects, which include antioxidant, anti-inflammatory, anti-mutagenic, hepatoprotective, cardioprotective, and neuroprotective properties (Kumar and Pandey, 2013; Panche et al., 2016).

Baicalein is one such flavonoid with a wide array of biological functions, including antioxidant (Kang et al., 2012), anti-inflammatory (Ren et al., 2021), antiviral (Moghaddam et al., 2014), anticancer (Cathcart et al., 2016), and cardioprotective (Zhao et al., 2016). It also inhibits acetylcholinesterase (Liao et al., 2022) and has neuroprotective properties (Sowndhararajan et al., 2018). 

Therefore, the purpose of this study was to explore the neuroprotective potential of combination therapy of baicalein with memantine in albino Wistar rats with aluminum chloride-induced neurotoxicity.

## 2 Material and methods

### 2.1 Drugs and chemicals

Memantine was obtained as a gift sample from Intas Pharmaceuticals (India). Baicalein was procured from the Chemical Centre (Mumbai, India). Aluminum chloride and acetylcholine iodide were purchased from Sigma Aldrich, USA. β-Amyloid antibodies (catalog no. SC-28365, lot no. I0117), and BDNF antibodies (catalog No.: SC-65514, lot no. L0816) were purchased from Santacruz Biotechnology Inc. (USA).

### 2.2 Experimental animals

Male albino Wistar rats weighing from 180 to 200 g were procured from the National Institute of Biosciences, Pune, India. Animals had unlimited access to a standard pellet diet and water. A week was spent acclimating the animals to the conditions prior to the start of the experiment. The study protocol was approved by the Institutional Animal Ethics Committee and was performed in accordance with the norms of the Committee for the Purpose of Control and Supervision of Experiments on Animals (CPCSEA), Government of India.

### 2.3 Experimental design

Rats were randomly divided into six experimental groups, each containing ten rats. Group I animals served as the normal control group, which received distilled water for 42 days. Group II was disease control and received aluminum chloride solution (100 mg/kg) for 42 days. Group III received aluminum chloride solution (100 mg/kg) and memantine (20 mg/kg) orally. Group IV received aluminum chloride solution (100 mg/kg) and baicalein (10 mg/kg) orally. Group V received an oral administration of aluminum chloride solution (100 mg/kg), memantine (20 mg/kg) and baicalein (5 mg/kg). Group VI was administered aluminum chloride solution (100 mg/kg), memantine (20 mg/kg) and baicalein (10 mg/kg) orally for 42 days. The dose of baicalein was selected based on the available literature (Zhou et al., 2016).

### 2.4 Behavioral assessment

#### 2.4.1 Locomotor Activity

A digital actophotometer (Inco, India) was used to assess the movement of each animal on days 21 and 42. Each animal was placed in an actophotometer equipped with infrared light-sensitive photocells for 5 min. Animal movement cuts off the light, which is recorded as a count. (Kumar et al., 2009; Auti and Kulkarni, 2019).

#### 2.4.2 Morris Water Maze (MWM) Test

The Morris Water Maze (MWM) test was performed as per the method reported in the literature (Morris, 1984; Auti and Kulkarni, 2019). The MWM consisted of a tank with a 150 cm diameter and 30 cm depth that was filled with water. The round maze was divided into four equal quadrants by marking the edges with tape. A sturdy platform was placed in one of the quadrants. Each animal was exposed to MWM in two phases: the acquisition phase, which lasted from days 17–20, and the retention phase, which lasted from days 21–42. During the acquisition phase, each animal was subjected to four trials with a 10-min gap, and every time it was placed in a different quadrant facing the wall. Each rat was given 120 s to explore the tank and find a platform. If the animal could not locate the platform, it was guided there and allowed to stay. The time taken by the animal to find the platform was recorded as acquisition latency. During the acquisition phase, the platform was visible to the animal, as it was placed 1 cm above the water level; however, during the retention phase, the water in the tank was made opaque, and the platform was placed 1 cm below the water level.

#### 2.4.3 Elevated Plus Maze (EPM) Test

The EPM test was conducted according to the method described in the literature (Walf and Frye, 2007; Auti and Kulkarni, 2019). The EPM had four arms, two open and two closed, connected by a central square. The arms measured 50 cm in length and 10 cm in width, and they were elevated 50 cm from the ground. EPM test was also performed in two phases: the acquisition phase was performed on day 20 when the initial transfer latency (ITL) was recorded, whereas the retention of memory was recorded on days 21 and 42 to record the 1st and 2nd transfer latency, respectively. During the experiment, the animal was placed in the open arm facing away from the central square, and the transfer latency was recorded as the time required for the animal to enter the closed arm. 

#### 2.4.4 Passive Avoidance (PA) Test

The Passive Avoidance (PA) apparatus had two chambers separated by a door; one chamber is illuminated by a lightbulb while the other was dark. The floor of the dark chamber was made of metal mesh attached to an electric supply for administering electric shock (40 V, 0.5 mA for 2 s). Each animal was subjected to two trials: acquisition and retention. The acquisition trial was conducted on day 20, and the retention trial on days 21 and 42. Each rat was placed in a lit chamber and allowed to explore the chamber for 60 s; after that, the door separating the two chambers was opened, and as the rat entered the dark chamber, an electric shock was administered. The rat was then transferred to its home cage. The time it took the animal to enter the dark chamber after the door was opened was recorded as pre-shock latency. The experiment was repeated on days 21 and 42 to record the post-shock latency (Prema et al., 2016; Auti and Kulkarni, 2019).

### 2.5 Brain dissection

At the end of the study, animals were euthanized by CO_2_ asphyxiation. The brain was extracted, rinsed with an ice-cold saline solution, and stored at −80°C.

The brain was removed from the skull and rinsed in ice-cold water to remove any surface blood. The brain was placed on a cold metal plate and separated into the right and left hemispheres. The olfactory bulb was removed, followed by the hippocampus. To expose the hippocampus, the ventral side of the brain was positioned upside down, and the midbrain was removed with the help of a spatula. Finally, the hippocampus was dissected from the cortex using two forceps, and the cortex and hippocampus were removed and stored in separately labeled containers at −80°C (Chiu et al., 2007).

Separated cortex and hippocampus were used for various biochemical and histopathological evaluations.

### 2.6 Biochemical assessment

#### 2.6.1 Measurement of oxidative stress parameters

Different oxidative stress parameters such as malondialdehyde (MDA), superoxide dismutase (SOD), catalase, and reduced glutathione (GSH) were measured in the hippocampus and cortex.

The separated hippocampus and cortex brain tissues were homogenized using a probe homogenizer (Polytron PT 2500E, Kinematica, Switzerland) in an ice-cold 0.1M phosphate buffer solution (pH 7.4). The MDA concentration was calculated as per the process described by Ohkawa et al. (1979). The SOD was measured using the method described by Paoletti et al. (1986). Catalase was determined using the method described by Luck (1971), and GSH was estimated as per the procedure by Ellman (1959).

#### 2.6.2 Measurement of acetylcholinesterase activity

The assessment of the acetylcholinesterase activity was carried out based on a previously published procedure (Pohanka et al., 2011).

### 2.7 Histopathological assessment

Following the completion of the behavioral assessment on day 42, animals were euthanized using CO_2_ asphyxiation for histopathological evaluation. Brains were removed, washed with an ice-cold isotonic normal saline, and immediately placed in 10% neutral buffered formalin (NBF). The hippocampus and cortex of each treatment group were evaluated separately for histopathological changes. These tissues were trimmed, and processing was done to dehydrate them in ascending alcohol concentrations, clear them in xylene, and embed them in paraffin wax. The Rotary Microtome was used to section paraffin-embedded tissue blocks to a thickness of 4–6 µm. Brain slices were stained with hematoxylin and eosin (H&E) stain and Congo red stain. The prepared slides were examined under a microscope to note histopathological lesions, if any. The severity of the observed lesions was recorded as No abnormality detected, Minimal (<1%), Mild (1%–25%), Moderate (26%–50%), Marked/Moderately Severe (51%–75%), Severe (76%–100%), and distribution was recorded as focal, multifocal, and diffuse.

### 2.8 Immunohistochemistry assessment (IHC)

For immunostaining, paraffin wax-embedded tissue blocks were sectioned at 4–6 µm thickness with a Rotary Microtome, placed on slides coated with Poly-L-Lysine, and incubated overnight at 4°C. Furthermore, these sections were deparaffinized, rehydrated, and incubated with citrate buffer at pH 6 in the decloaking chamber. Slides were incubated in a 3% hydrogen peroxide block for 20 min to inhibit endogenous peroxidase. The primary antibody was a β-Amyloid (b-4) antibody, and the secondary antibody was peroxidase-labeled anti-rabbit IgG. The staining was visualized by a reaction with diaminobenzidine color reagent and then counterstained with hematoxylin. Finally, the sections were rinsed with Tris-buffered saline (TBS), dehydrated in alcohol, and cleared in xylene before mounting using DPX. All the sections were examined under a light microscope to record the intensity of the antigen-antibody reaction.

The procedure was identical for immunohistochemical assay of BDNF expression, except primary antibodies used were pro-BDNF-5H8. The slides were observed under a photographic microscope at 400X.

### 2.9 Statistical analysis

The statistical analysis was performed using GraphPad Prism V8.0 for Windows. Behavioral parameters were evaluated using a two-way ANOVA (analysis of variance) and the Bonferroni test with a level of significance of *p* < 0.05. The oxidative stress parameters, AChE activity, and immunohistochemistry optical density data were evaluated using a one-way ANOVA using Dunnett’s test at a level of significance of *p* < 0.05. The experimental data were expressed as mean ± standard error of the mean (SEM).

## 3 Results

### 3.1 Behavioral assessment

#### 3.1.1 Actophotometer

**FIGURE 1:**
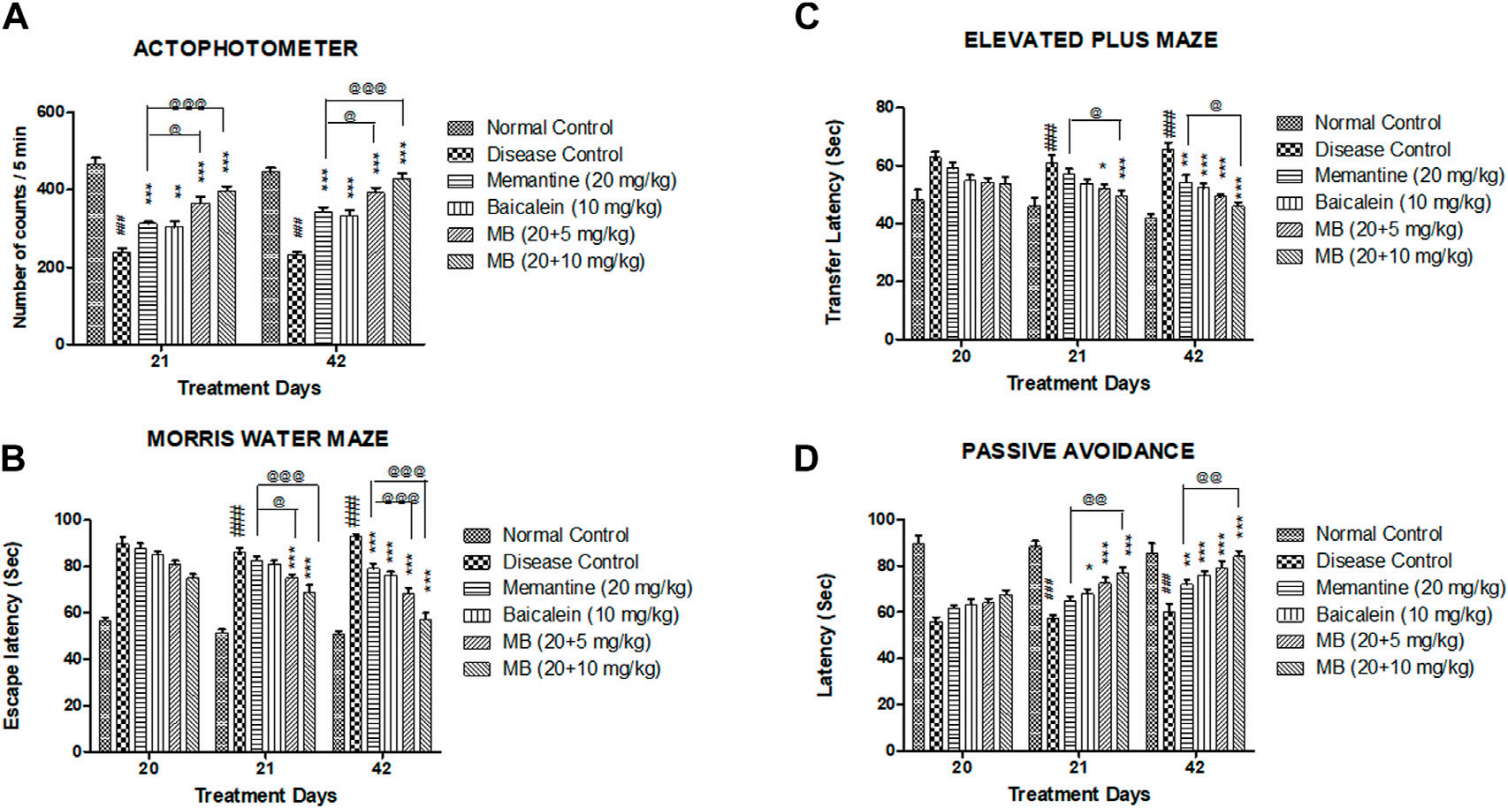
Effects of baicalein and baicalein with memantine on behavioral assessment. **(A)** Actophotometer, **(B)** Morris Water Maze, **(C)** Elevated Plus Maze, **(D)** Passive Avoidance. Data are expressed as mean ± SEM (*n* = 6), with ###*p* < 0.001 when compared to the normal control group, *p < 0.05, **p < 0.01, and ***p < 0.001 when compared to the disease control group. @*p* < 0.05, @@*p* < 0.01, @@@*p* < 0.001 when compared with memantine treatment group. SEM: Standard error of the mean. MB: Memantine + baicalein.

The disease control group indicated decreased locomotor activity as compared to the normal control group. Compared with the disease control group, locomotor activity was significantly increased in both treatment groups of baicalein with memantine on days 21 and 42 (*p* < 0.001). The locomotor activity of animals treated with baicalein and memantine was significantly greater than that of animals treated with memantine alone (20 mg/kg) (Figure 1A).

#### 3.1.2 Morris Water Maze test

The disease control group exhibited significantly increased escape latency (*p* < 0.001) when compared to the normal control group. Memantine administered with baicalein at doses of 20 + 5 and 20 + 10 mg/kg significantly decreased escape latency (*p* < 0.001) when compared with the disease control group. The escape latency was significantly reduced in animals treated with baicalein and memantine when compared with animals treated with memantine alone (20 mg/kg) (Figure 1B).

#### 3.1.3 Elevated Plus Maze test

In the EPM test, a substantial increase (*p* < 0.001) in transfer latency was observed in the disease control group when compared to the normal control group. Memantine administered with baicalein at doses of 20 + 5 and 20 + 10 mg/kg significantly decreased the transfer latency when compared with the disease control group. The transfer latency was significantly decreased (*p* < 0.05) in animals treated with baicalein and memantine when compared with animals treated with memantine alone (20 mg/kg) (Figure 1C).

#### 3.1.4 Passive avoidance test

The post-shock latency was significantly decreased (*p* < 0.001) in the disease control group when compared to the normal control group. Compared with the disease control group, treatment of baicalein with memantine at doses of 20 + 5 mg/kg and 20 + 10 mg/kg considerably increased post-shock latency (*p* < 0.001). The post-shock latency was significantly greater in animals treated with baicalein and memantine than in animals treated with memantine alone (20 mg/kg) (Figure 1D).

### 3.2 Effect of oxidative stress parameters

Compared with the normal control group, the hippocampus and cortex tissue of the disease control group exhibited a significant increase in MDA levels (*p* < 0.001) and a significant reduction in levels of SOD, catalase, and GSH (*p* < 0.001). The treatment of baicalein with memantine showed a significant decrease in the levels of MDA and an increase in the levels of SOD, catalase, and GSH in the hippocampus and cortex when compared with the disease control group. (Table 1; Table 2).

**TABLE 1:** Effect of baicalein and baicalein with memantine on oxidative stress parameters in hippocampus.

Group	MDA (nmol/mg of protein)	SOD (µmol/mg of protein)	CAT (nmol of H_2_O_2_ decomposed/min/ mg of protein)	GSH (µmol/mg of protein)
Normal control	7.70 ± 0.50	8.65 ± 0.47	9.68 ± 0.62	11.34 ± 071
Disease control	13.58 ± 1.81^###^	4.83 ± 0.27^###^	3.69 ± 0.70^###^	5.09 ± 0.85^###^
Memantine (20 mg/kg)	10.02 ± 0.34^∗^	6.08 ± 0.40	4.13 ± 0.22	5.56 ± 0.66
Baicalein (10 mg/kg)	9.48 ± 0.50^∗∗^	6.63 ± 0.52	6.03 ± 0.55^∗∗^	6.41 ± 0.82
Memantine (20 mg/kg) + Baicalein (5 mg/kg)	8.95 ± 0.51^∗∗^	7.08 ± 0.57^∗^	7.14 ± 0.20^∗∗∗^	8.20 ± 0.82^∗^
Memantine (20 mg/kg) + Baicalein (10 mg/kg)	8.39 ± 0.27^∗∗∗^	7.54 ± 0.70^∗∗^	7.79 ± 0.03^∗∗∗^	9.49± 0.83^∗∗^

Data are expressed as mean ± SEM (*n* = 6), ###*p* < 0.001 when compared with normal control, ∗*p* < 0.05, ∗∗*p* < 0.01, ∗∗∗*p* < 0.001 when compared with disease control group. SEM: Standard Error of Mean.

**TABLE 2:** Effect of baicalein and baicalein with memantine on oxidative stress parameters in cortex.

Group	MDA (nmol/mg of protein)	SOD (µmol/mg of protein)	CAT (nmol of H_2_O_2_ decomposed/min/ mg of protein)	GSH (µmol/mg of protein)
Normal control	6.79 ± 0.65	8.21 ± 0.37	8.85 ± 0.75	10.21 ± 0.78
Disease control	11.15 ± 0.61^###^	4.47 ± 0.26^###^	3.61 ± 0.13^###^	4.45 ± 0.58^###^
Memantine (20 mg/kg)	8.95 ± 0.46^∗^	5.08 ± 0.55	5.73 ± 0.21^∗∗^	5.06 ± 0.51
Baicalein (10 mg/kg)	8.67 ± 0.36^∗∗^	6.16 ± 0.22	6.04 ± 0.29^∗∗∗^	6.32 ± 0.92
Memantine (20 mg/kg) + Baicalein (5 mg/kg)	8.41 ± 0.52^∗∗^	6.91 ± 0.49^∗∗^	7.01 ± 0.32^∗∗∗^	7.62 ± 1.02^∗^
Memantine (20 mg/kg) + Baicalein (10 mg/kg)	7.94 ± 0.36^∗∗∗^	7.72 ± 0.64^∗∗∗^	8.29 ± 0.16^∗∗∗^	8.93 ± 0.77^∗∗^

Data are expressed as mean ± SEM (*n* = 6), ###*p* < 0.001 when compared with normal control, ∗*p* < 0.05, ∗∗*p* < 0.01, ∗∗∗*p* < 0.001 when compared with disease control group. SEM: Standard Error of Mean.

### 3.3 Acetylcholinesterase activity

**FIGURE 2:**
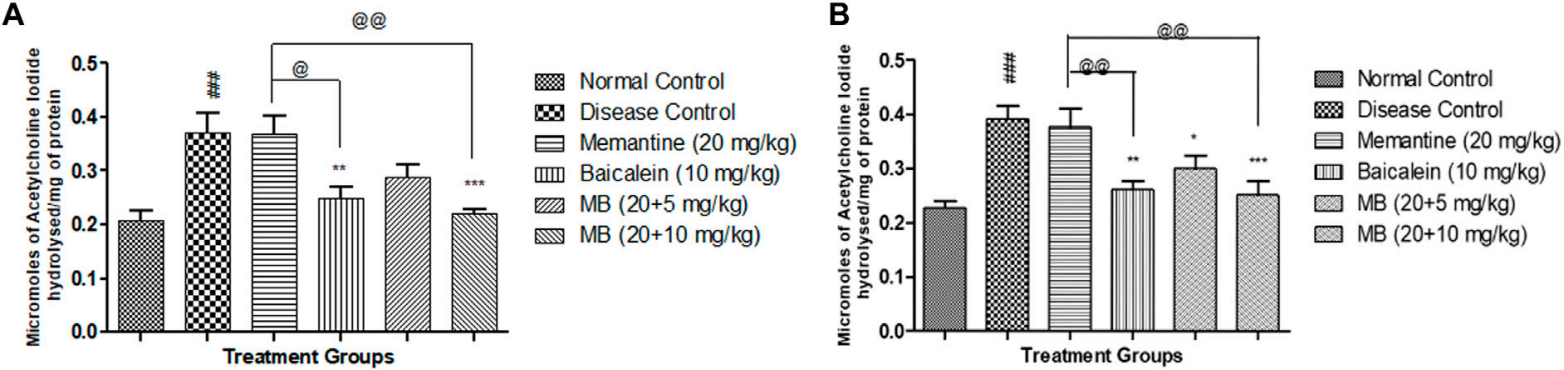
Effects of baicalein and baicalein with memantine on AChE Activity **(A)**. *Hippocampus*, **(B)**. Cortex. Data are expressed as mean ± SEM (*n* = 6), with ###*p* < 0.001 when compared to the normal control group, *p < 0.05, **p < 0.01, and ***p < 0.001 when compared to the disease control group, @*p* < 0.05, @@*p* < 0.01 when compared to the memantine treatment group. SEM: Standard Error of the Mean.

The disease control group’s AChE activity was significantly higher than the normal control group’s. Treatment of memantine with baicalein at doses of 20 + 10 mg substantially reduced AChE activity in the cortex and hippocampus when compared with the disease control group. Cortex and hippocampus AChE activity was considerably decreased in animals treated with baicalein and memantine at doses of 20 + 10 mg when compared with animals treated with memantine alone (20 mg/kg) (Figures 2A, B).

### 3.5 Histopathological examination

#### 3.5.1 Haematoxylin and eosin staining

Microscopic examination of H&E-stained brain sections was used to evaluate the morphological changes between various treatment groups. Animal brains were evaluated histopathologically for viable neurons or loss of structure of neurons, which involves loss of integrity of cellular cytoplasm and nuclei, and is considered neurodegeneration. Microphotographs of viable neurons revealed a condensed layer of neuronal cells with vesicular nuclei, whereas hyperchromic neuronal lesions revealed obvious alterations in the cell layers and organelles that directed neurodegeneration.

**FIGURE 3:**
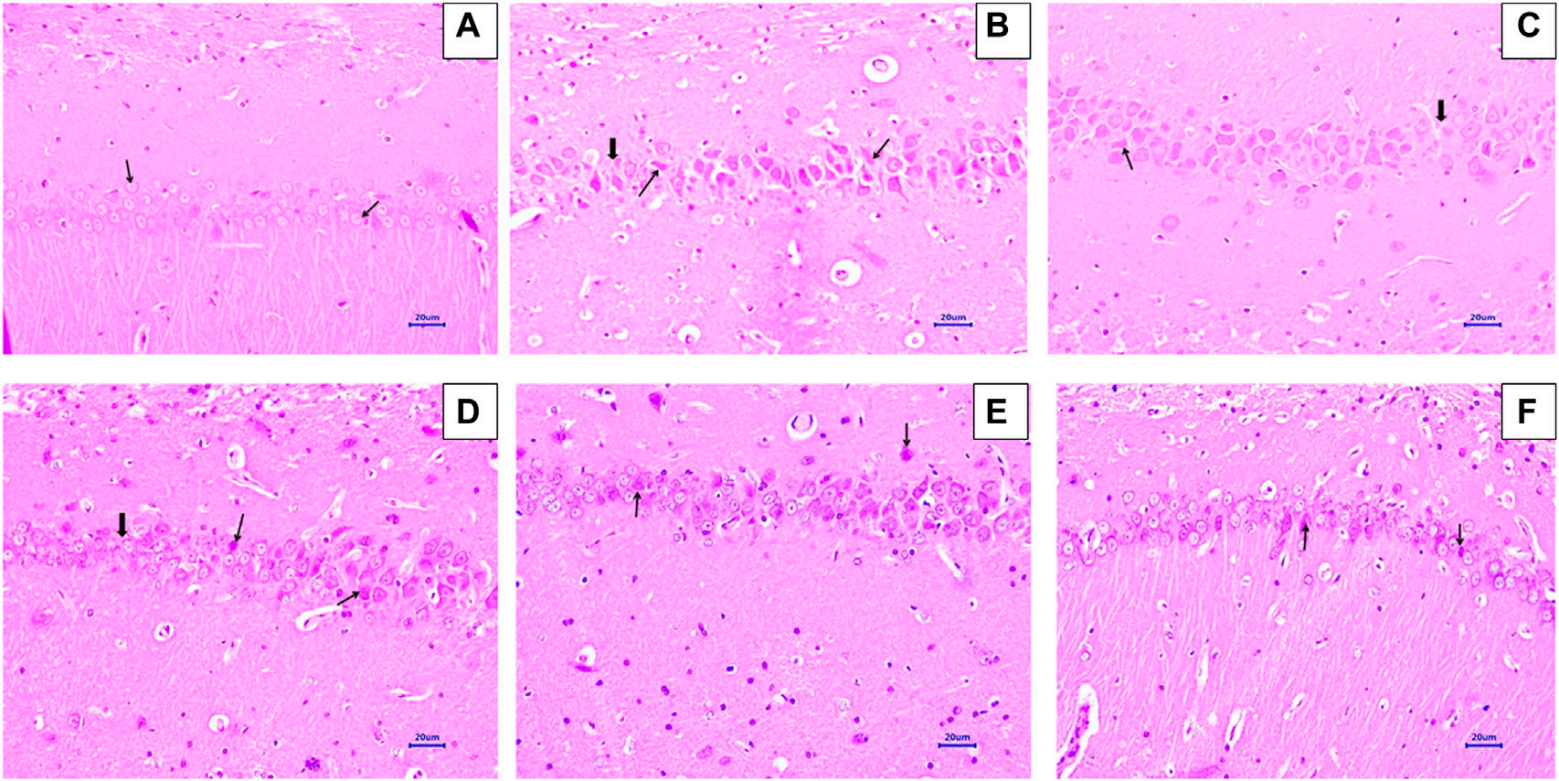
Effects of baicalein and baicalein with memantine on H&E-stained hippocampus tissue (400X), **(A)** Normal control: showing normal histology and normal neuronal cells in the hippocampus (small arrow) **(B)** Disease control: hippocampus neuronal degeneration with pyknotic nuclei (small arrow), hippocampus neuronal cell layer reduction (large arrow) **(C)** Memantine (20 mg/kg): hippocampus neuronal degeneration with pyknotic nuclei (small arrow), hippocampus neuronal cell layer reduction (large arrow) **(D)** Baicalein (10 mg/kg): hippocampus neuronal degeneration with pyknotic nuclei (small arrow), hippocampus neuronal cell layer reduction (large arrow) **(E)** Memantine + baicalein (20+5 mg/kg): hippocampus neuronal degeneration with pyknotic nuclei (small arrow) **(F)** Memantine + baicalein (20 + 10 mg/kg): hippocampus neuronal degeneration with pyknotic nuclei (small arrow).

**FIGURE 4:**
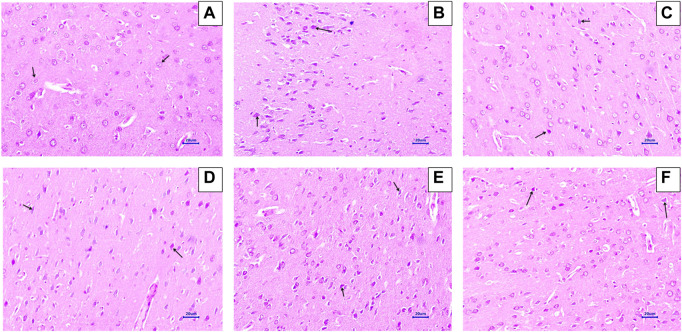
Effects of baicalein and baicalein with memantine on H&E-stained cortex tissue (400X), **(A)** Normal control: showing normal histology and normal neuronal cells in the parietal cortex (small arrow) **(B)** Disease control: showing neuronal degeneration with pyknotic nuclei in the cortex (small arrow) **(C)** Memantine (20 mg/kg): showing normal histology, normal neuronal cells in the parietal cortex (small arrow), **(D)** Baicalein (10 mg/kg): showing neuronal degeneration with pyknotic nuclei in the cortex (small arrow), **(E)** Memantine + baicalein (20+5 mg/kg): showing neuronal degeneration with pyknotic nuclei in the cortex (small arrow), **(F)** Memantine + baicalein (20 + 10 mg/kg): showing neuronal degeneration with pyknotic nuclei in the cortex (small arrow).

Microscopic examination of hippocampus and cortex sections from animals in the normal control group did not show signs of any pathologically significant lesions. However, animals from the disease control group showed multifocal, moderate-to-marked neuronal degeneration in the hippocampus and cortex. Standard drug-treated (memantine at doses of 20 mg/kg) animals showed multifocal, minimal-to-mild neuronal degeneration in the cortex and hippocampus and a focal mild reduced layer of neuronal cells in the hippocampus. Animals administered with baicalein (10 mg/kg) exhibited multifocal minimal-to-mild neuronal degeneration in the hippocampus and cortex, in addition to a focal, minimal-to-mild reduced layer of neuronal cells in the hippocampus. Treatment of baicalein with memantine at doses of 20 + 5 mg/kg showed multifocal, minimal-to-mild neuronal degeneration in the hippocampus and cortex. Furthermore, the treatment of baicalein with memantine at doses of 20 + 10 mg/kg showed focal minimal-to-mild neuronal degeneration in the hippocampus and cortex (Figure 3, 4).

#### 3.5.2 Congo red staining

**FIGURE 5:**
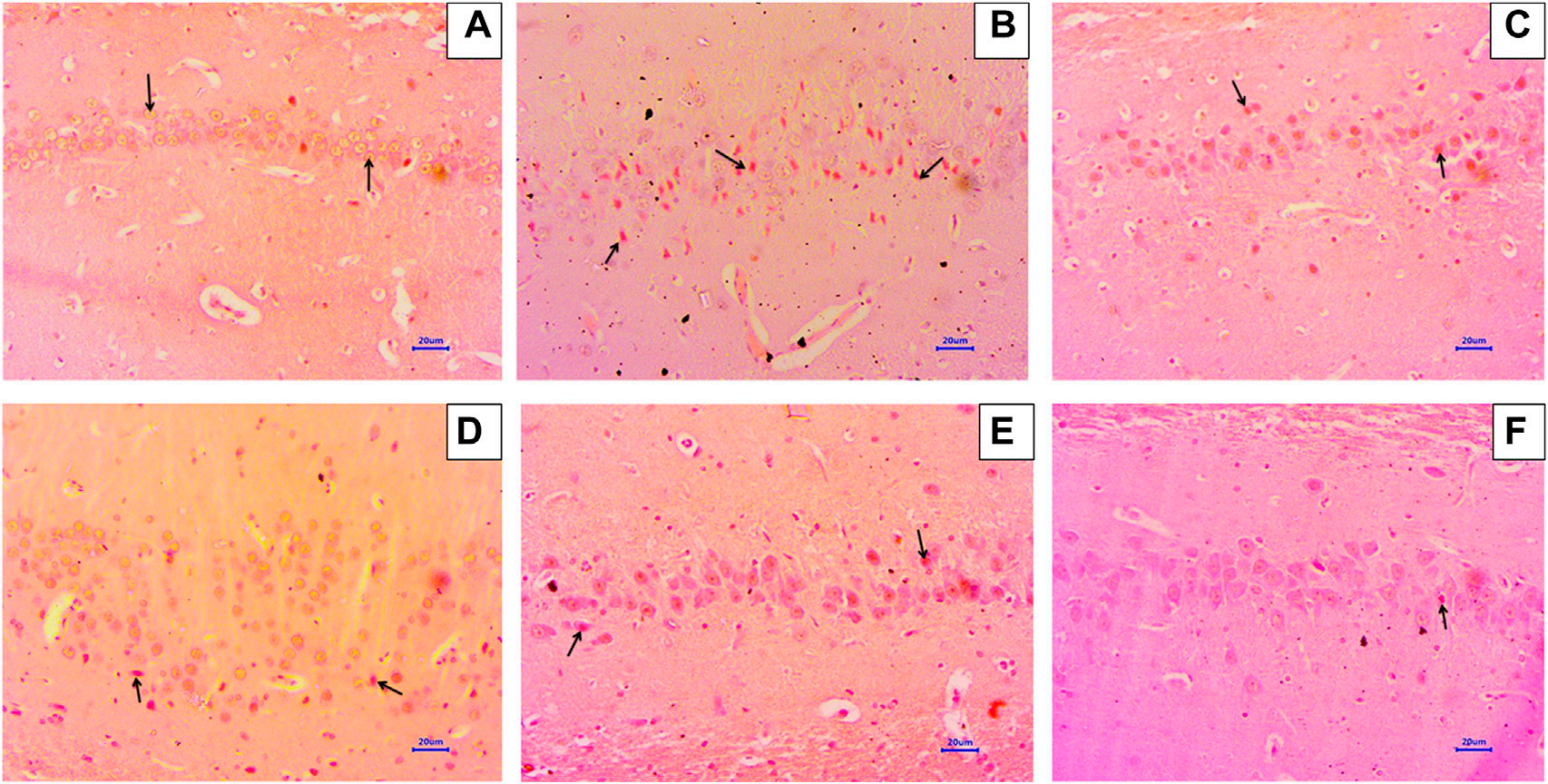
Effects of baicalein and baicalein with memantine on Congo red staining of the hippocampus tissue (400X), **(A)** Normal control: showing normal histology, normal neuronal cells in the hippocampus (small arrow), **(B)** Disease control: showing β-Amyloid deposition (small arrow) in the hippocampus, **(C)** Memantine (20 mg/kg): showing β-Amyloid deposition (small arrow) in the hippocampus, **(D)** Baicalein (10 mg/kg): showing β-Amyloid deposition (small arrow) in the hippocampus, **(E)** Memantine + baicalein (20+5 mg/kg): showing β-Amyloid deposition (small arrow) in the hippocampus, **(F)** Memantine + baicalein (20 + 10 mg/kg): showing β-Amyloid deposition (small arrow) in the hippocampus.

**FIGURE 6:**
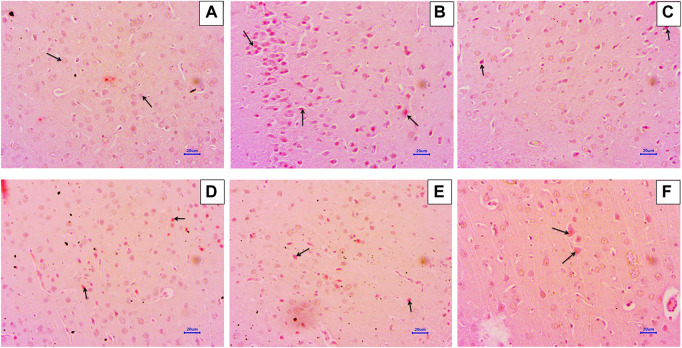
Effects of baicalein and baicalein with memantine on Congo red staining of the cortex tissue (400X), **(A)** Normal control: showing normal histology, normal neuronal cells in the parietal cortex (small arrow), **(B)** Disease control: showing β-Amyloid deposition (small arrow) in the parietal cortex, **(C)** Memantine (20 mg/kg): showing β-Amyloid deposition (small arrow) in the parietal cortex, **(D)** Baicalein (10 mg/kg): showing β-Amyloid deposition (small arrow) in the parietal cortex, **(E)** Memantine + Baicalein (20+5 mg/kg): showing β-Amyloid deposition (small arrow) in the parietal cortex, **(F)** Memantine + baicalein (20 + 10 mg/kg): showing β-Amyloid deposition (small arrow) in the parietal cortex.

Microscopic examination of the hippocampus and cortex sections stained with Congo red from the normal control group did not show deposition of amyloid. However, animals in the disease control group had multifocal, moderate-to-severe amyloid deposition in the hippocampus and cortex. Animals treated with memantine at doses of 20 mg/kg showed minimal-to-mild multifocal impairment in the hippocampus and cortex. Animals treated with baicalein at doses of 10 mg/kg showed multifocal, mild-to-moderate amyloid deposition in the hippocampus and cortex. Furthermore, animals treated with 20 + 5 mg/kg of baicalein and memantine showed multifocal mild amyloid deposition in the hippocampus and cortex. Similarly, baicalein and memantine treatment at doses of 20 + 10 mg/kg resulted in focal, minimal-to-mild amyloid deposition in the hippocampus and cortex (Figures 5, 6).

### 3.6 Immunohistochemistry assessment (IHC)

#### 3.6.1 β-Amyloid immunohistochemistry assessment 

**FIGURE 7:**
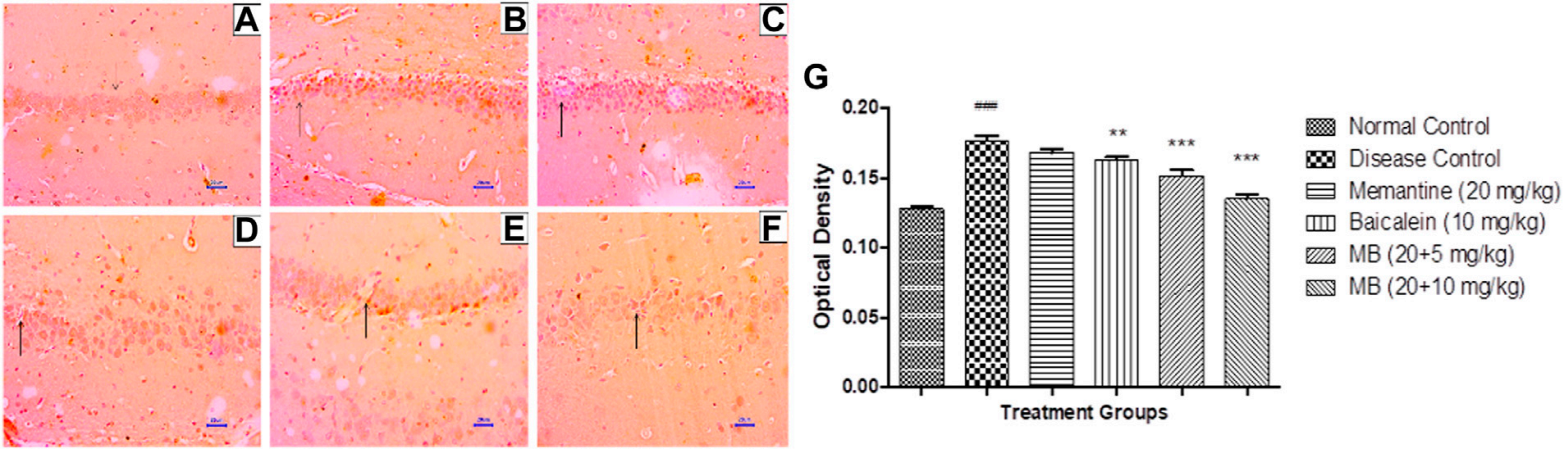
Effects of baicalein and baicalein with memantine on β-Amyloid expression in hippocampus tissue (400X), **(A)** Normal control: showing normal neuronal cells without immunoreactivity at the hippocampus (small arrow), **(B)** Disease control: showing moderately enhanced β-Amyloid expression as evidenced by brown coloration in the hippocampus (small arrow), **(C)** Memantine (20 mg/kg): showing mildly enhanced β-Amyloid expression as evidenced by brown coloration in the hippocampus (small arrow), **(D)** Baicalein (10 mg/kg): showing mildly enhanced β-Amyloid expression as evidenced by brown coloration in the hippocampus (small arrow), **(E)** Memantine + baicalein (20+5 mg/kg): showing mildly enhanced β-Amyloid expression as evidenced by brown coloration in the hippocampus (small arrow), **(F)** Memantine + baicalein (20 + 10 mg/kg): showing mildly enhanced β-Amyloid expression as evidenced by brown coloration in the hippocampus (small arrow), **(G)** OD data for ICH. Data are expressed as mean ± SEM, with ###*p* < 0.001 when compared to the normal control group, **p < 0.01, and ***p < 0.001 when compared to the disease control group. AlCl_3_, Aluminum chloride; SEM, Standard error of the mean; IHC, Immunohistochemistry.

**FIGURE 8:**
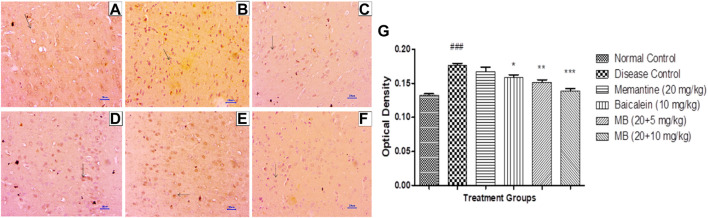
Effects of baicalein and baicalein with memantine on β-Amyloid expression in cortex tissue (400X), **(A)** Normal control: showing normal neuronal cells without immunoreactivity at the cortex (small arrow), **(B)** Disease control: showing moderately enhanced β-Amyloid expression as evidenced by the brown coloration of cortical neurons (small arrow), **(C)** Memantine (20 mg/kg): showing mildly enhanced β-Amyloid expression as evidenced by the brown coloration of cortical neurons (small arrow), **(D)** Baicalein (10 mg/kg): showing moderately enhanced β-Amyloid expression as evidenced by the brown coloration of cortical neurons (small arrow), **(E)** Memantine + baicalein (20+5 mg/kg): showing mildly enhanced β-Amyloid expression as evidenced by the brown coloration of cortical neurons (small arrow), **(F)** Memantine + Baicalein (20 + 10 mg/kg): showing mildly enhanced β-Amyloid expression as evidenced by the brown coloration of cortical neurons (small arrow), **(G)** OD data for ICH. Data are expressed as mean ± SEM, with ###*p* < 0.001 when compared to the normal control group, and *p < 0.05, **p < 0.01, and ***p < 0.001 when compared to the disease control group. AlCl_3_, Aluminum chloride; SEM, Standard error of the mean; IHC, Immunohistochemistry.

Immunohistochemistry (IHC) analysis of hippocampus and cortex sections from animals in the normal control group showed no β-Amyloid deposition. However, microscopic examination of tissues from the disease control group revealed moderate-to-marked β-Amyloid expression in the hippocampus and cortex. Animals treated with memantine at doses of 20 mg/kg revealed mildly increased β-Amyloid expression in the hippocampal and cortical regions of the brain. Furthermore, animals treated with baicalein (10 mg/kg) showed mildly enhanced β-Amyloid expression in the cortex and mildly to moderately enhanced β-Amyloid expression in the hippocampus. Treatment of baicalein and memantine at doses of 20 + 5 mg/kg resulted in mildly enhanced β-Amyloid expression in the cortex and mild to moderately enhanced β-Amyloid expression in the hippocampus. Moreover, treatment with memantine and baicalein at doses of 20 + 10 mg/kg showed mildly enhanced β-Amyloid expression in the hippocampus and cortex (Figures 7, 8).

#### 3.6.2 Immunohistochemistry assessment for BDNF

**FIGURE 9:**
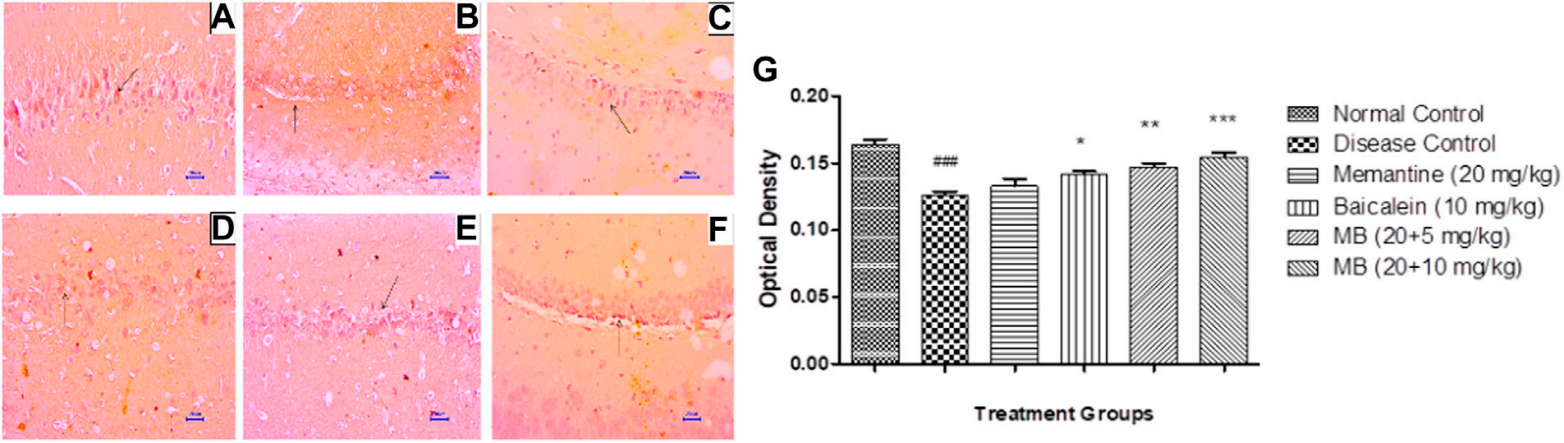
Effects of baicalein and baicalein with memantine on BDNF expression in hippocampus tissue (400X), **(A)** Normal control: showing moderately enhanced BDNF expression, as evidenced by the brown coloration of hippocampus neurons (small arrow), **(B)** Disease control: showing mild BDNF expression, as evidenced by brown coloration at *Hippocampus* neurons (small arrow), **(C)** Memantine (20 mg/kg): showing moderately enhanced BDNF expression, as evidenced by the brown coloration of hippocampus neurons (small arrow), **(D)** Baicalein (10 mg/kg): showing mild BDNF expression, as evidenced by brown coloration of hippocampus neurons (small arrow), **(E)** Memantine + baicalein (20+5 mg/kg): showing moderately enhanced BDNF expression, as evidenced by brown coloration of hippocampus neurons (small arrow), **(F)** Memantine + baicalein (20 + 10 mg/kg): showing moderately enhanced BDNF expression, as evidenced by the brown coloration of hippocampus neurons (small arrow), **(G)** OD data for ICH. Data are expressed as mean ± SEM, with ###*p* < 0.001 when compared to the normal control group, and *p < 0.05, **p < 0.01, and ***p < 0.001 when compared to the disease control group. AlCl_3_, Aluminum chloride; SEM, Standard error of the mean; BDNF, Brain-derived neurotrophic factor; IHC, Immunohistochemistry.

**FIGURE 10:**
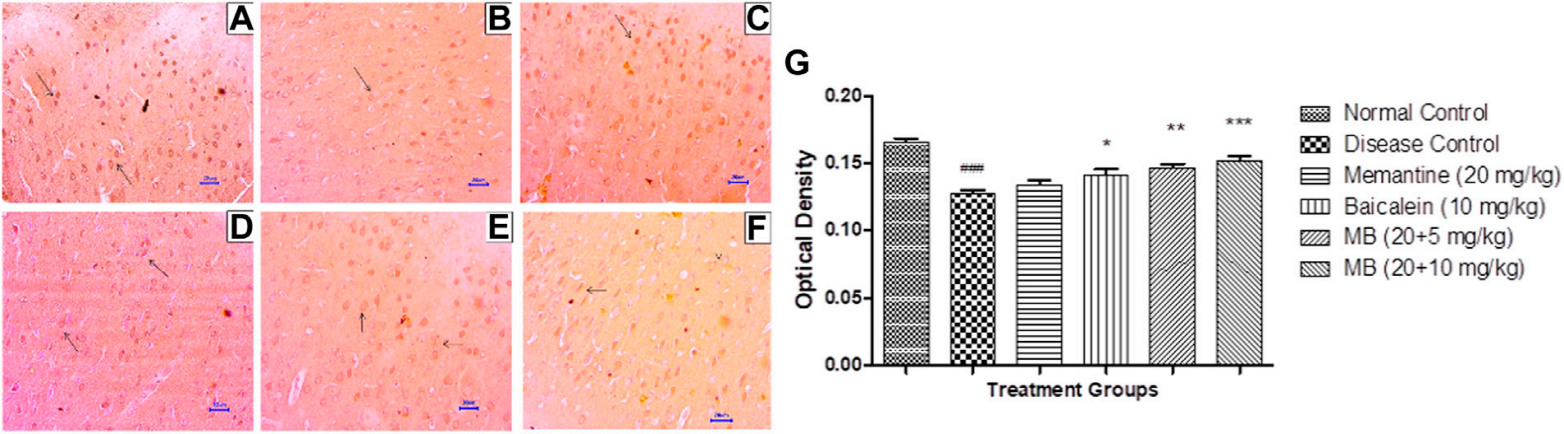
Effects of baicalein and baicalein with memantine on BDNF expression in cortex tissue (400X), **(A)** Normal control: showing moderately enhanced BDNF expression, as evidenced by brown coloration in cortical neurons (small arrow), **(B)** Disease control: showing reduced BDNF expression, as evidenced by brown coloration in cortical neurons (small arrow), **(C)** Memantine (20 mg/kg): showing moderately enhanced BDNF expression, as evidenced by brown coloration in cortical neurons (small arrow), **(D)** Baicalein (10 mg/kg): showing mild BDNF expression, as evidenced by brown coloration in cortical neurons (arrow), **(E)** Memantine + Baicalein (20+5 mg/kg): showing mildly enhanced BDNF expression, as evidenced by brown coloration in cortical neurons (small arrow), **(F)** Memantine + baicalein (20 + 10 mg/kg): showing moderately enhanced BDNF expression, as evidenced by brown coloration in cortical neurons (small arrow), **(G)** OD data for ICH. Data are expressed as mean ± SEM, with ###*p* < 0.001 when compared to the normal control group, *p < 0.05, **p < 0.01, and ***p < 0.001 when compared to the disease control group. AlCl_3_, Aluminum chloride; SEM, Standard error of the mean; BDNF, Brain-derived neurotrophic factor; IHC, Immunohistochemistry.

Immunohistochemistry (IHC) evaluation in animals from the disease control group showed decreased BDNF expression in the hippocampus and cortex. Treatment with memantine (20 mg/kg) showed mildly to moderately enhanced BDNF expression in the cortical and hippocampal regions of the brain. Baicalein treatment (10 mg/kg) revealed mild BDNF expression in the hippocampus and mildly to moderately enhanced BDNF expression in the cortex. Treatment of baicalein with memantine at doses of 20 + 5 mg/kg showed mildly to moderately BDNF-enhanced expression in the cortex and hippocampus. Moreover, baicalein with memantine at doses of 20 + 10 mg/kg showed moderately enhanced BDNF expression in both the hippocampus and cortex (Figures 9, 10).

## 4 Discussion

In 2020, 9.3% of the population was 65 or older, and the number is expected to increase to 16% in 2050 (World Population Ageing, 2020). This increase in the elderly population is predicted to have a considerable social and economic impact on the healthcare infrastructure.

Aluminum is the most prevalent metal and has been documented to be neurotoxic to animals (Exley, 1999). When it enters the brain, it can cause topological changes within the DNA (Bharathi et al., 2003). Aluminum exacerbates brain oxidative damage, causes inflammation, and induces Aβ deposition. Al can also induce oxidative damage by associating with negatively charged brain phospholipids, which can be attacked by reactive oxygen species (ROS) such as O_2_^˙-^, H_2_O_2,_ and OH. Al can also stimulate iron-initiated lipid peroxidation in the Fenton reaction, which causes ROS production and Fe^3+^ formation (Yuan et al., 2012).

Furthermore, Al gains access to the brain *via* the specific high-affinity receptors for transferrin (TfR) (Kumar et al., 2009). Chronic aluminum administration has been reported to impair the glutamate NO-cGMP pathway in the cerebellum, leading to memory impairment and neurobehavioral deficits. It is also a potent cholinotoxin that causes cholinergic dysfunction (Gulya et al., 1990; Canales et al., 2001).

In addition, Al exposure has been shown to boost Aβ_(1–42)_ expression, leading to an increased Aβ production in the animal brain *via* activation of the APP, β-secretase (BACE1), and γ-secretase (presenilin-1) mRNA transcription, which is also linked to a decrease of α-secretase proteins (ADAM9, ADAM10, and ADAM17) (Liang et al., 2013).

Studies have shown that aluminum may lead to the disruption of proinflammatory cytokines, resulting in a decrease in BDNF expression (Johnson and Sharma, 2003). Therefore, the present study was designed with an animal model of aluminum chloride-induced neurotoxicity to determine the potential effect of baicalein and baicalein in combination with memantine by evaluating the behavioral, biochemical, and histopathological parameters in the brain.

In β-Amyloid precursor protein-deficient mice, there is decreased locomotor activity and forelimb grip strength, demonstrating compromised neuronal and or muscular function (Zheng et al., 1995). In the present study, significant impairment in locomotor activity was observed in the disease control group. Treatment of baicalein with memantine at doses of 20 + 10 mg/kg improved locomotor activity in experimental animals. Similar results were observed by Minkeviciene et al. for memantine treatment, which improved locomotor activity in a transgenic mouse model (Minkeviciene et al., 2004); also, the study performed by Xiong et al. for baicalein demonstrated the improvement in locomotor activity as compared to the control group (Xiong et al., 2011).

The Morris Water Maze test was used to assess learning and spatial memory acquisition (Bromley-Brits et al., 2011). In the present study, a reduction in escape latency in the treatment group of baicalein with memantine at doses of 20 + 10 mg/kg suggests a recovery from neurotoxicity induced by AlCl_3_ in the brain.

Minkeviciene et al. and Van Dam et al. also demonstrated improved learning and spatial memory acquisition after treatment with memantine (Minkeviciene et al., 2004; Van Dam et al., 2005). In the MWM study, Zhuang et al. (2013) emphasized the beneficial effect of baicalein on cognitive function. In the present study, an increase in escape latency in the treatment group of baicalein in combination with memantine at doses of 20 + 10 mg/kg suggests a recovery from neurotoxicity induced by AlCl_3_ in the brain.

The EPM test was used to measure transfer latency, which helped to understand spatial memory and learning. Chronic AlCl_3_ exposure reduced open-arm exploration and increased transfer latency, expressing anxiogenic properties (Murugaiyan and Bhargavan, 2021). The results of the study show that treatment with baicalein in combination with memantine at doses of 20 + 10 mg/kg improved transfer latency. The passive avoidance test was deemed a useful tool for assessing learning, memory, and cognition. A decrease in post-shock latency time after AlCl_3_ administration was an indication of progressive memory impairment (Lakshmi et al., 2015). The results of the present study indicate an increase in post-shock latency in the treatment group of baicalein with memantine at a 20 + 10 mg/kg dose. The improvement in post-shock latency was also observed in the memantine treatment group by Gorbachenko and Lukyanetz (2020) and the baicalein-treated group by Wong et al. (2014). Those findings are in line with our study results.

The administration of 100 mg/kg AlCl_3_ orally for 42 days resulted in a significant increase in oxidative stress markers, leading to neurodegeneration through lipid peroxidation. MDA is one such oxidative stress marker that causes reduced axonal mitochondrial turnover and disruption of the Golgi apparatus (Al-Amin et al., 2019). By combining baicalein with memantine, the levels of lipid peroxidation in the hippocampus and cortex were significantly reduced. SOD is considered a frontline defense mechanism and plays a critical role in catalyzing highly reactive free radicals (O_2_-) into hydrogen peroxide (H_2_O_2_) and molecular oxygen (Younus, 2018). A prominent rise in SOD levels as compared to the disease control group was observed in the hippocampal and cortical regions in the treatment group of baicalein with memantine (20 + 10 mg/kg). Catalase is another prime antioxidant enzyme involved in the conversion of hydrogen peroxide into water and oxygen. The results showed a decrease in catalase activity in the AlCl_3_-treated disease control group, which is consistent with previously published studies (Aboelwafa et al., 2020). Treatment of baicalein with memantine (20 + 10 mg/kg) improved catalase levels in both the hippocampus and cortex. The GSH system is required for the cellular detoxification of reactive oxygen species (Elshamy et al., 2021). The combination of baicalein and memantine significantly increased cellular GSH levels. Thus, an improvement in levels of the antioxidant enzyme system when treated with baicalein and memantine revealed its strong antioxidant activity in AlCl_3_-induced neurotoxicity. The current findings agreed with those of a previous study conducted by Shanta et al. (2020), in which memantine improved SOD and GSH levels while decreasing MDA levels, emphasizing its antioxidant effect and free radical scavenging ability. Furthermore, studies have indicated that, in addition to scavenging free radicals, baicalein is also able to increase antioxidant capabilities by recovering the activities of endogenous antioxidant enzymes and up-regulating their gene expression, which supports the results of the present study (Liu et al., 2007; Lee et al., 2011; Takeshima et al., 2011; Kang et al., 2012).

Acetylcholine (ACh) is a neurotransmitter involved in both the central and peripheral nervous systems, supporting cognitive and physiological functions within the body. Acetylcholinesterase (AChE) is a cholinergic enzyme involved in the termination of neuronal transmission and synaptic signaling. Changes in AChE activity are indicative of learning and cognitive disabilities. The present study indicates that treatment of baicalein with memantine at doses of 20 + 10 mg/kg significantly reduced the AChE activity in the hippocampus and cortex when compared with the disease control group, which supports its use in the management of AD. In numerous previous *in vitro* studies, baicalein has shown substantial AChE inhibitory activity compared to other flavonoids (Balkis et al., 2015; Han et al., 2019), and the results are in line with our study.

β-Amyloid plaques are a hallmark of the pathogenesis of AD. Excessive deposition of Aβ induces neuronal dysfunction and synaptic coordination. The administration of AlCl_3_ to experimental animals for 42 days enhanced the expression of β-Amyloid in the hippocampus and cortex. The combination of baicalein and memantine has been shown to reverse the formation of β-Amyloid plaques formation caused by AlCl_3_ administration. The results are consistent with earlier studies where memantine reduced Aβ_1-42_ production at sub-toxic concentrations in rat primary cortical cultures. Also, in APP/PS1 transgenic mice, memantine has significantly reduced brain levels of Aβ_1-42_ (Alley et al., 2010; Ito et al., 2017). Similarly, previous studies have demonstrated that baicalein protects against amyloid-β induced neuronal cell lesions, both *in vitro* and *in vivo* (Gasiorowski et al., 2012; Yu et al., 2012; Shi et al., 2021; Wong et al., 2014. These findings support the Aβ ameliorating effect of baicalein. The study conducted by Shi et al. (2021) also corroborates the results of the present research.

The expression of BDNF is a key factor in determining neuronal health. In the present investigation, BDNF deficiency was evident in the AlCl_3_-treated group. The combination of baicalein and memantine enhanced BDNF expression in the hippocampus and cortex. In 2021, Shi et al. found similar results, with improved BDNF expression regulating PDE2A/PDE4-dependent neuroprotective pathways, which ultimately improve neuronal remodeling and cognitive function.

Histopathological assessment of the brain using H&E and Congo red staining suggests that treatment with AlCl_3_ in rats induces degenerative lesions and β-Amyloid plaque deposition in the hippocampus and cortex. There were no signs of pathological lesions and low levels of β-Amyloid deposition in the baicalein with memantine treatment group, which indicates a neuroprotective effect against AlCl3-induced neurotoxicity in albino Wistar rats. Numerous earlier studies have observed histopathological changes in the hippocampus and cortex due to AlCl_3_ (100 mg/kg), and our study has confirmed this neurotoxic effect (Thenmozhi et al., 2015). On the contrary, histopathological studies have indicated that baicalein inhibits morphological changes, which is consistent with our research (Liu et al., 2007).

To summarize, the current study demonstrates that baicalein with memantine significantly improves the cognitive deficits and neuropathological changes induced by AlCl_3_ in albino Wistar rats.

## 5 Conclusion

The results of this study indicate that the treatment of baicalein with memantine may be useful in the management of Alzheimer’s disease.

## Data Availability

The raw data supporting the conclusion of this article will be made available by the authors, without undue reservation.
